# Ionic Selectivity and Permeation Properties of Human PIEZO1 Channels

**DOI:** 10.1371/journal.pone.0125503

**Published:** 2015-05-08

**Authors:** Radhakrishnan Gnanasambandam, Chilman Bae, Philip A. Gottlieb, Frederick Sachs

**Affiliations:** State University of New York at Buffalo, Department of Physiology and Biophysics, Buffalo, New York, United States of America; Dalhousie University, CANADA

## Abstract

Members of the eukaryotic *PIEZO* family (the human orthologs are noted *hPIEZO1* and *hPIEZO2*) form cation-selective mechanically-gated channels. We characterized the selectivity of human PIEZO1 (hPIEZO1) for alkali ions: K^+^, Na^+^, Cs^+^ and Li^+^; organic cations: TMA and TEA, and divalents: Ba^2+^, Ca^2+^, Mg^2+^ and Mn^2+^. All monovalent ions permeated the channel. At a membrane potential of -100 mV, Cs^+^, Na^+^ and K^+^ had chord conductances in the range of 35–55 pS with the exception of Li^+^, which had a significantly lower conductance of ~ 23 pS. The divalents decreased the single-channel permeability of K^+^, presumably because the divalents permeated slowly and occupied the open channel for a significant fraction of the time. In cell-attached mode, 90 mM extracellular divalents had a conductance for inward currents carried by the divalents of: 25 pS for Ba^2+^ and 15 pS for Ca^2+^ at -80 mV and 10 pS for Mg^2+^ at -50 mV. The organic cations, TMA and TEA, permeated slowly and attenuated K^+^ currents much like the divalents. As expected, the channel K^+^ conductance increased with K^+^ concentration saturating at ~ 45 pS and the K_D_ of K^+^ for the channel was 32 mM. Pure divalent ion currents were of lower amplitude than those with alkali ions and the channel opening rate was lower in the presence of divalents than in the presence of monovalents. Exposing cells to the actin disrupting reagent cytochalasin D increased the frequency of openings in cell-attached patches probably by reducing mechanoprotection.

## Introduction

PIEZO channels are cation selective and the largest membrane proteins with 32–34 transmembrane domains and they function as oligomers [1, 2]. Only recently identified, the basic properties of this family of channels have not yet been extensively studied compared to those of other non-selective cation channels of the nicotinic acetylcholine, glutamate, purinergic or serotonin receptor families [3]. Although members of the PIEZO family show sequence similarity amongst themselves, their sequences do not resemble those of other cation-selective channels [1].

PIEZO channels transiently over-expressed in HEK293 cells are activated either by stretching patches or by indenting the cell in whole-cell recordings. Using whole-cell currents, Coste et al. showed that both monovalent and divalent cations permeate mouse Piezo (MmPiezo) channels [1]. Whole-cell recordings may not be consistent with single-channel measurements due to differences in solutions on the cytoplasmic face [4–6]. The reported slope conductance of the MmPiezo1 channel in cell-attached mode is approximately 23 pS with Cs^+^ as the dominant ion in the pipette and the cells in a Na^+^ bath saline. Although some single-channel permeation properties have been reported, these measurements did not provide quantitative information on ion selectivity and conductance. A basic knowledge of ion permeation, including those of divalents such as Ca^2+^ and Mg^2+^, is crucial to understanding channel function *in situ* as well as providing guidance for *in vitro* experiments.

We studied the selectivity and permeability of both monovalent and divalent ions in human PIEZO1 (hPIEZO1) channels over-expressed in HEK293 cells. The conductance of these channels to monovalent alkali ions was highest for K^+^, intermediate and similar for Cs^+^ and Na^+^ and lowest for Li^+^ yielding a conductance sequence K^+^ > Cs^+^ ≅ Na^+^ > Li^+^. Divalent ions except for Mn^2+^ permeated the channel but more slowly than the monovalents and they also reduced K^+^ currents as expected for these less permeant competitors. Both TMA and TEA permeated the channel and like the divalents reduced K^+^ current. These data suggest the narrowest region of the pore was ~ 8 A˚ in diameter.

## Materials and Methods

### Electrophysiology

#### Cell Culture and Transfection Protocols

HEK293 cells were cultured at 37°C for 1 day after passage. Recordings were performed on cells that were transiently transfected with 200 ng of *hPIEZO1* cDNA. Transfections were performed using TransIT 293 reagent from Mirus Bio LLC. Recordings were performed 1–2 days after transfection.

#### Single-Channel Recordings

Currents were activated by applying suction to patches in the cell-attached configuration or with positive pressure for the outside-out configuration. Pressure steps were applied to the pipette using a high speed pressure clamp [7] (ALA Scientific). All experiments were performed at room temperature. Our bath solution contained (in mM): 150 KCl, 10 HEPES, 1 MgCl_2_, 1 CaCl_2_ at pH 7.4, which clamped the resting membrane potential to zero. The pipette solutions for monovalent conductance measurements contained (in mM): 150 KCl / 150 NaCl / 150 CsCl / 150 LiCl, 10 HEPES, pH 7.4. The solutions were pH adjusted with the respective hydroxides. Pipette solutions for divalent ion titration experiments on K^+^ currents contained 150 KCl, 10 HEPES and divalent ions (Mg^2+^, Ca^2+^ or Ba^2+^) at concentrations of 10μM, 100 μM, or 1mM. For recordings of currents carried by pure divalents, we used 90 mM MgCl_2_, CaCl_2_ or BaCl_2_ in the pipette saline with 10 mM HEPES at pH 7.4. The pH of 90 mM Mn^2+^ solutions was adjusted to 7.4 with a small amount of Tris. For testing the permeability of organic ions, the pipette solution contained (in mM): 150 TMA or TEA, 10 HEPES and the pH was adjusted to 7.4 with Tris. To examine the effect of organic ions on K^+^ we used mixed solutions of K^+^ and TMA or TEA (in mM): 150 KCl, and 20–150 mM TMA/TEA adjusted to pH 7.4 with KOH. The junction potentials were highest when using 90 mM divalents or pure 150 mM TMA / TEA in the pipette solution. The junction potentials calculated (using pClamp10 junction potential calculator) for 90 mM Ba^2+^, Ca^2+^ and Mg^2+^ were -7.8 mV, -8.3 mV and -9.1 mV respectively. S1 Table in the supporting information summarizes the compositions of the bath saline and the pipette saline, the recording configuration and the junction potential expected for that configuration.

#### Permeability Ratio Calculations

Monovalent currents were obtained from outside-out patches.The bath solution contained 150 KCl, 10 HEPES, 1 MgCl_2_, 1 CaCl_2_ at pH 7.4 and the pipette solutions contained 150 KCl / NaCl / 150 CsCl / 150 LiCl, 10 HEPES at pH 7.4. The reversal potential for each ion was determined from its current-voltage relationship and corrected for the junction potential such that V_R_ = V_P_ – V_L_ (where V_R_, V_P_ and V_L_ are the reversal potential, command potential and the junction potential, respectively). The permeability ratios with K^+^ as the reference were calculated using the simplified Goldman-Hodgkin-Katz (GHK) equation for monovalent permeant ions: $E_{rev}=\frac{RT}{zF}\ln\frac{P_{K}{\left[{\text{K}}^{+}\right]}_{o}}{P_{X}{\left[{\text{X}}^{+}\right]}_{i}}$

#### Treatment of Cells with Cytochalasin D

To increase single-channel activity, cells in the bath solution were treated with 2 μM cytochalasin D and incubated at room temperature for 15 min before commencing recordings. One or two cells were used from each dish.

#### Analysis

Digitized data were analyzed with QuB Express software (www.qub.buffalo.edu) [8]. Regions containing channel openings were selected and binned to generate all-point histograms which were fit to Gaussians to measure the open channel amplitudes. The slope conductance was defined as the slope of a linear fit of the mean current amplitudes (weighted by inverse of the variance) versus voltage. Chord conductances and slope conductances were calculated from data pooled from multiple cells. The conductance (*γ*_*s*_) versus [K^+^] data were fit to Michaelis-Menten equation, $\gamma_{s}=\frac{\gamma_{\max}}{1+\frac{K_{D}}{\left[K^{+}\right]}}$, where *K*_*D*_ represents the [K^+^] at half-maximal conductance.

## Results

### Single-Channel Monovalent Selectivity

**Fig 1:**
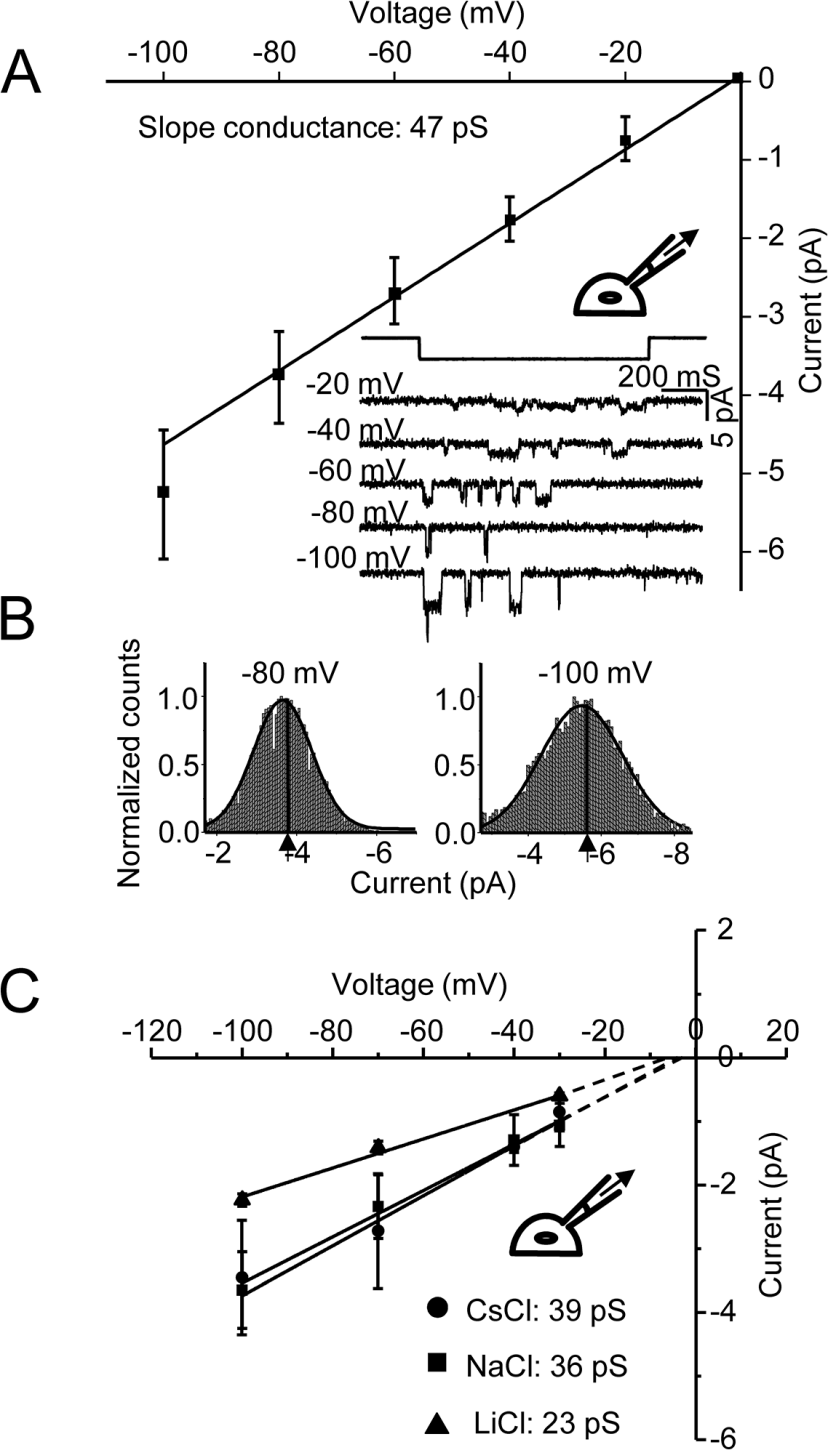
Extracellular K^+^ currents through hPIEZO1 channels. (A) Unitary currents from *hPIEZO1* cDNA transfected cells in response to pressure steps (pipette suction) applied via a cell-attached pipette containing K^**+**^. A linear fit of the I/V relationship yielded a slope conductance of 47 pS (n = 6). (B) Amplitude histograms of the unitary open channel current for the indicated voltages (n = 6). The calculated chord conductance was 47 pS at -80 mV and 53 pS at -100 mV. (C) I/V relationships for monovalent ions Cs^**+**^ (n = 3), Na^**+**^ (n = 6) and Li^**+**^ (n = 5); the linear fits were extrapolated to intersect the abscissa. K^**+**^ is our standard for comparison. The unitary current with Cs^**+**^ and Na^**+**^ were lower than that with K^**+**^. With Li^**+**^ as the current carrying ion the unitary current was approximately halved.

The current-voltage relationship for K^+^ in cell-attached patches was fairly linear and reversed near 0 mV as expected. The slope conductance was 47 pS (Fig 1A). Fig 1A (inset) shows a typical record of unitary K^+^ currents from a multi-channel patch in the cell-attached configuration. The mean currents at -80 mV and -100 mV are shown in the all-point histograms (Fig 1B). Fitting the peaks with Gaussians, the chord conductance of K^+^ at -80 mV and -100 mV were ~47 pS and ~53 pS, respectively. The other alkali ions Na^+^, Cs^+^ and Li^+^, had I/V data similar to that of K^+^ with slope conductances of 39 pS for Cs^+^, 36 pS for Na^+^ and 23 pS for Li^+^ (Fig 1C).

### Monovalent Ion Permeability and Effect of K^+^ Concentration on Conductance

**Fig 2:**
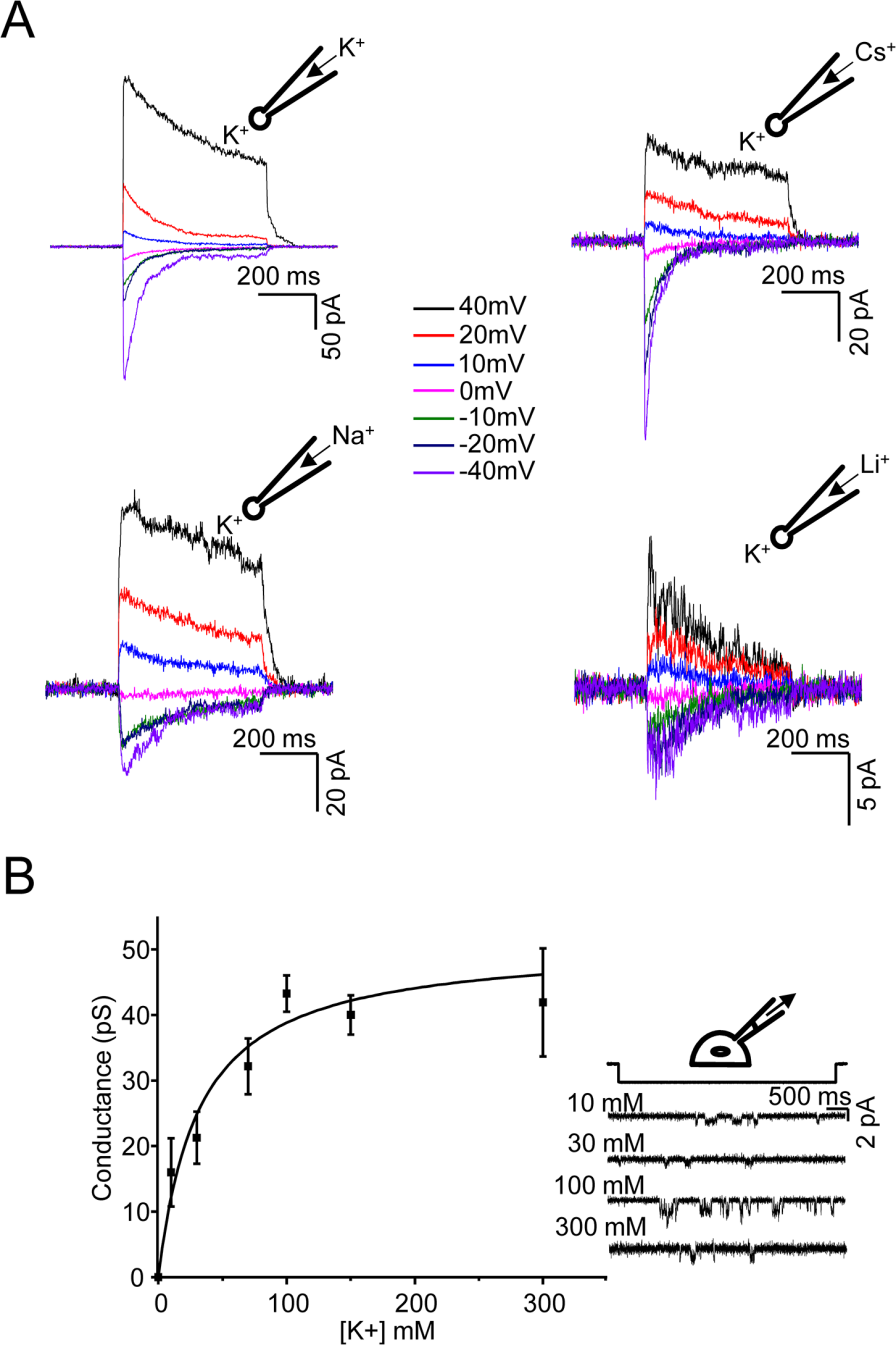
Permeability ratio of monovalent ions and the relationship between [K^+^] and conductance. (A) Permeability ratios were determined by using the Goldman-Hodgkin-Katz (GHK) equation for monovalent cations. I/V relationships were obtained from outside-out patches with Cs^**+**^, Na^**+**^ or Li^**+**^ on the cytoplasmic side and K^**+**^ on the extracellular side. The reversal potentials were compensated *a posteriori* for the liquid junction potentials resulting from assymetric ion composition across the outside-out patch. The monovalent pipette solutions did not contain Ca^**2+**^ or Mg^**2+**^ ions. (B) Conductance as a function of K^**+**^ ion concentration ([K^**+**^]) in cell-attached patches (n = 3 patches for each concentration). The pipette solutions did not contain Ca^**2+**^ or Mg^**2+**^. The comparison across concentrations was performed for a fixed driving force of 40 mV. The conductance versus [K^**+**^] data were curve-fitted using the Michealis-Menten equation: $\gamma_{s}=\frac{\gamma_{\max}}{1+\frac{K_{D}}{\left[K^{+}\right]}}$. The junction potentials were ignored because they were within 0–2 mV. Single channel traces corresponding to 10 mM, 30 mM, 100 mM and 300 mM are shown in the inset.

We estimated the permeability ratio of Cs^+^, Na^+^ and Li^+^ using K^+^ as the reference ion in outside-out patches (Fig 2A). The applied potentials were corrected *a posteriori* for junction potentials such that V_m_ = V_P_ – V_L_ (where V_P_ and V_L_ are the command potential and junction potential, respectively). The resulting permeability ratios were 1:0.88:0.82:0.71 (P_K_:P_Cs_:P_Na_:P_Li_). The permeability values of Na^+^ and Cs^+^ were very close to each other implying that the pore is large enough so that the hydrated radii of the two ions are almost indistinguishable and that the pore does not require dehydrated ions.

The conductance of channels generally increases with the concentration of permeant ions. We examined the relationship between conductance and the concentration of K^+^ (10mM–300mM) in cell-attached patches (Fig 2B); the unitary currents are shown in the inset. We defined the conductance as *g* = *i / (V*_*m*_ *-V*_*rev*_ *)* where *V*_*m*_ is the membrane potential, *V*_*rev*_ is the reversal potential and *i* is the unitary current. For a driving force of 40 mV *(V*_*m*_ *-V*_*rev*_ *)*, the conductance (*g*) was ~16–20 pS with 10–30 mM [K^+^]. The mean conductance *g* increased to 43 pS at 100–300 mM [K^+^] (with a 40 mV driving force). We could not record with higher concentrations of K^+^ as they disrupted the patch. The relationship between [K^+^] and single-channel conductance was fit to the Michaelis-Menten equation yielding a maximal conductance of 51 pS and the K_D_ of K^+^ for the channel of ~32 mM (Fig 2B).

### hPIEZO1 Channels in Patches Have a Significant Divalent Permeability

**Fig 3:**
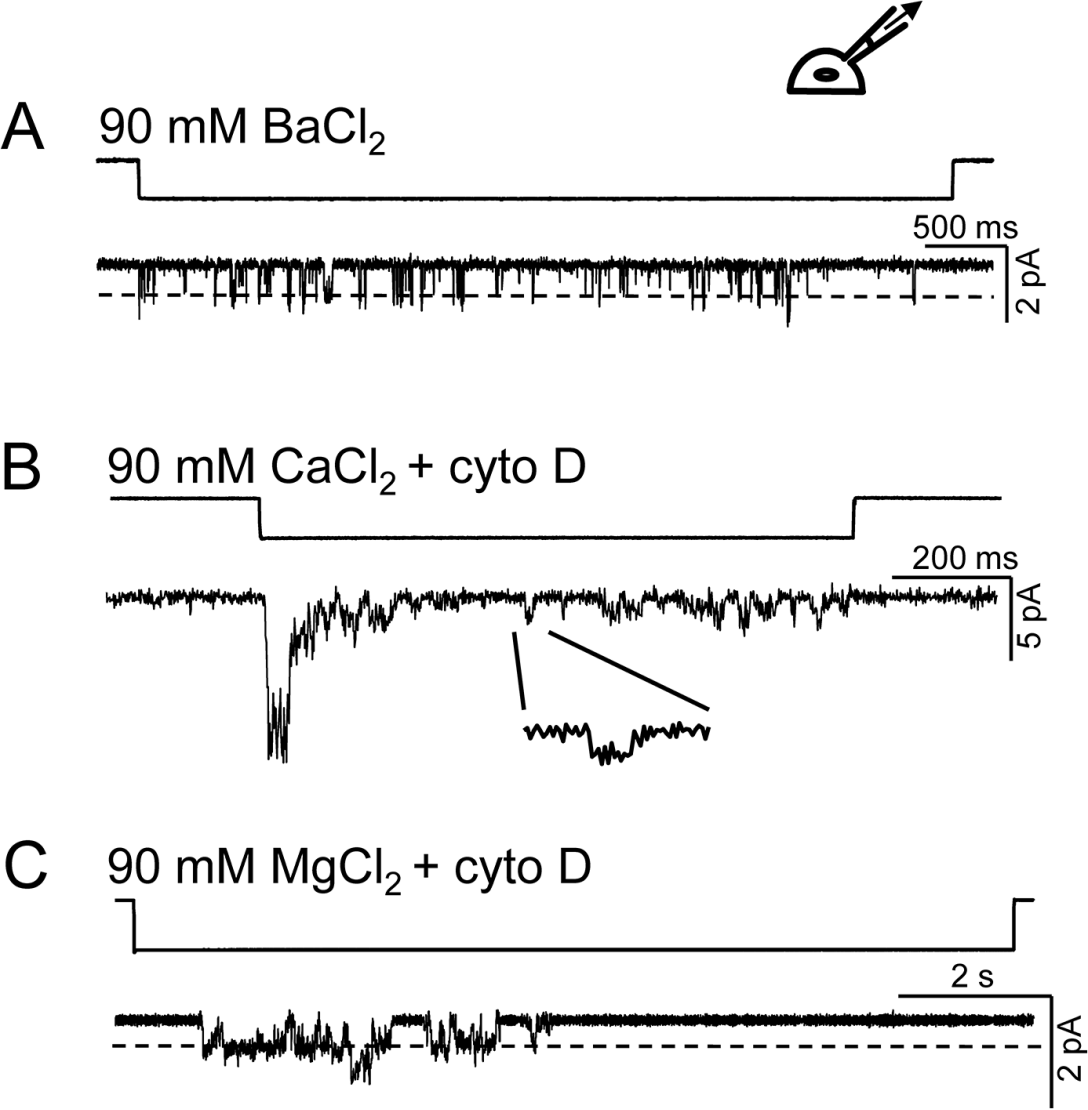
Divalent ions traverse hPIEZO1 channels. (A) Currents from channels activated in response to suction in a cell-attached patch with 90 mM extracellular Ba^**2+**^ held at -80 mV (n = 10). (B) Current from a cell (pre-exposed to cytochalasin D) showing multiple channels activated by suction with 90 mM extracellular (in the pipette) Ca^**2+**^ (n = 10). The inset shows a single opening from which the calculated conductance was ~15 pS at -80 mV. (B) The conductance of the channel using 90 mM extracellular Mg^**2+**^ was ~10 pS at -50 mV (n = 4). Correcting for the junction potentials (-7.8 mV, -8.3 mV and -9.1 mV for 90 mM Ba^**2+**^, Ca^**2+**^ and Mg^**2+**^, respectively) would cause a minor increase in the chord conductance.

We examined whether the divalent ions: 90 mM Ba^2+^, Ca^2+^ and Mg^2+^, can traverse the channel and produce currents in the absence of monovalent ions. Several non-selective cation channels including hPIEZO1 are permeable to divalents [1, 9–13]. In cell-attached patches, the chord conductance of the unitary currents with extracellular Ba^2+^ at -80 mV was ~25 pS (Fig 3A). Channel opening rates were slower with extracellular divalents than with monovalents with the exception of Ba^2+^. This behavior suggests an allosteric effect of divalents on the gating kinetics. To facilitate channel activation in cell-attached patches when using high concentrations of divalents, we pre-exposed cells to 2 μM cytochalasin D (Fig 3B and 3C) to disrupt the cytoskeleton that was shunting stresses around the channels (mechanoprotection).

### Cytochalasin D Treatment Facilitates Divalent Ion Permeability

Emphasizing a critical role played by the channels’ environment, cytochalasin D (cyto D) treatment slowed the activation kinetics in whole-cell recordings but not in cell-attached patches [14]. We suspect that cyto D, which prevents actin polymerization, enhances activation of the channels by reducing mechanoprotection that normally shields the channel from stress [15]. The mechanoprotective effect itself is emphasized by the fact that we cloned the human form of *hPIEZO1* from HEK cells that generally exhibit a low level of intrinsic mechanosensitive channel activity [16, 17]. We used cyto D to increase the probability of observing activity in cells over-expressing *hPIEZO1*. We frequently observed channel openings with high concentration of divalents following cyto D treatment. The chord conductances of Ca^2+^ and Mg^2+^ were ~15 pS at -80mV and ~10 pS at -50mV, respectively. We did not observe Mn^2+^ currents even with cyto D treatment. However, it was possible to elicit outward current (K^+^ currents) from the cell-attached patches at positive membrane potentials suggesting that the blocking Mn^2+^ could be displaced by an efflux of K^+^.

### Inhibition of K^+^ Currents by Divalent Ions

**Fig 4:**
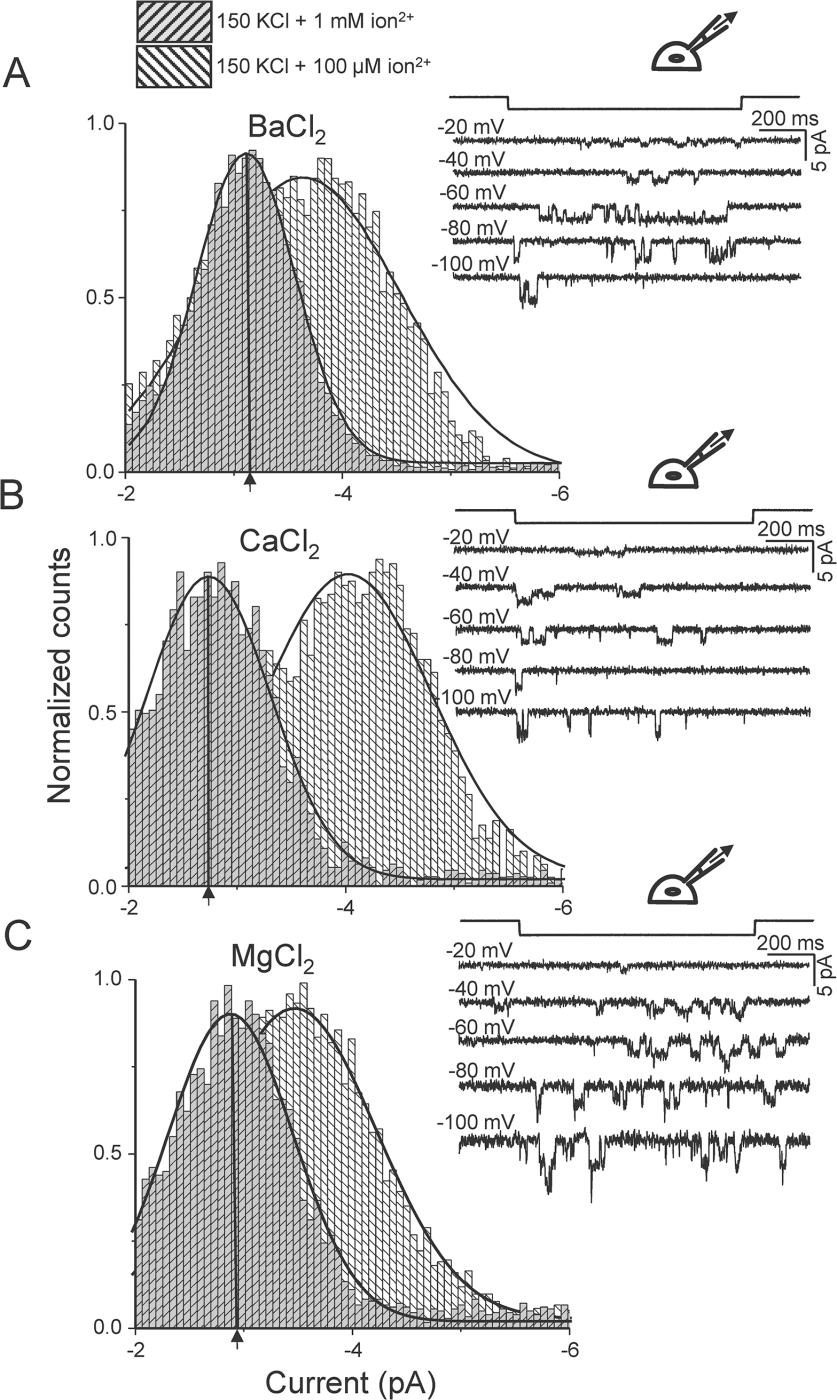
K^+^ unitary current amplitude is reduced by 1 mM divalent ions. (A) Unitary currents in the presence of 1 mM Ba^**2+**^ in the pipette with K^**+**^ saline (right). There is a leftward shift in the mean current amplitude when the concentration of Ba^**2+**^ is increased from 100 μM to 1 mM as shown by the amplitude histogram (at -80 mV). A similar shift in unitary current is observed with an increase in Ca^**2+**^ (B) and with an increase in Mg^**2+**^ (C). Three patches with multiple openings were analyzed for each concentration.

**Fig 5:**
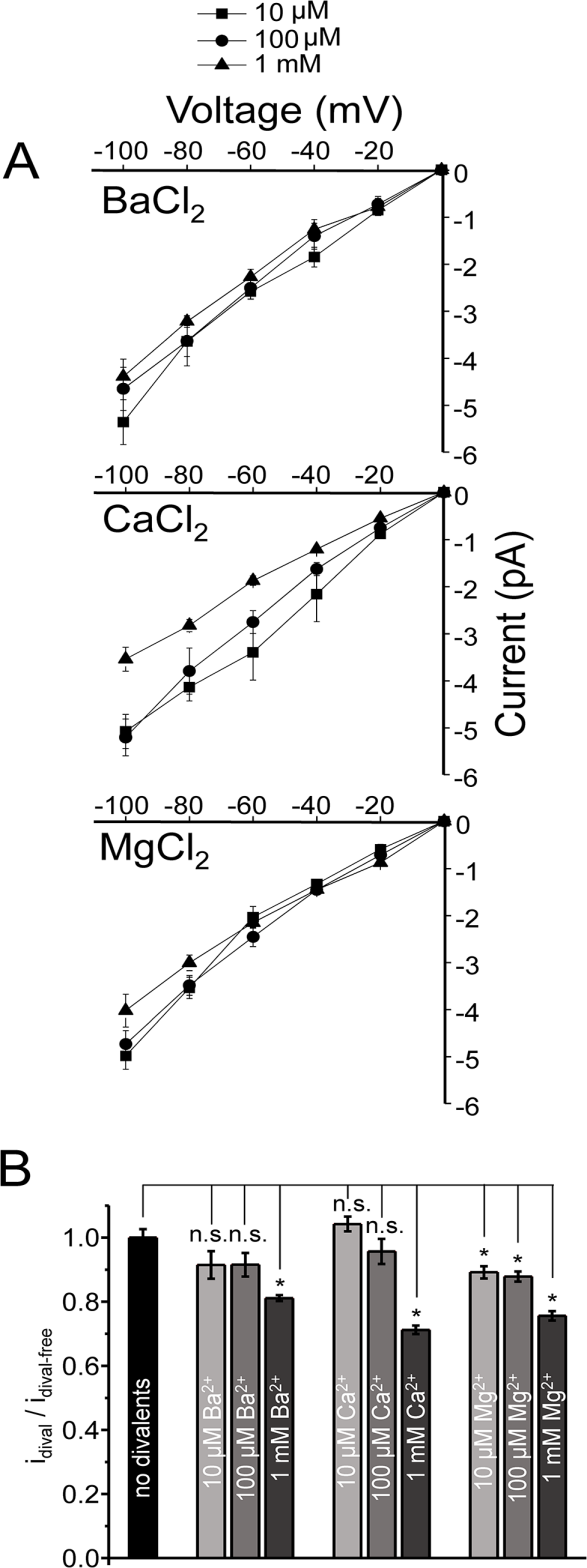
Current-Voltage (I/V) relationships with a mix of divalents and K^+^ ions. I/V plots for mixtures of K^+^ with Ba^2+^ (A), Ca^2+^ (B) or Mg^2+^ (C) in the pipette saline of cell-attached patches. Three concentrations (squares: 10 μM, circles: 100 μM and triangles: 1 mM) were used for each divalent ion used in the mixture (D) Unitary amplitude in the presence of each divalent (i_dival_) has been normalized to that of pure K^**+**^ current in divalent-free pipette solution (i_dival-free_) at -80 mV. Student’s t-tests were performed to examine if reductions in current amplitudes were statistically significant (*: P < 0.01; n.s.: not significant). The reduction of unitary K^**+**^ current is greatest with Ca^**2+**^. The junction potentials were in the range of 0.3–0.5 mV for 1 mM Ba^**2+**^, Ca^**2+**^ or Mg^**2+**^ (highest concentration) included in the pipette solution.

Extracellular divalents (Mg^2+^, Ca^2+^ and Ba^2+^) reduced inward K^+^ currents in a concentration- and voltage-dependent manner. The reduction in single-channel current varied among the ions tested, but for all divalents a concentration of 1mM inhibited K^+^ currents. At -80mV, the current carried by K^+^ alone was -3.9 ± 0.1 pA (Fig 1B). Adding 1 mM Mg^2+^ to the K^+^ based pipette solution (the extracellular solution) reduced K^+^ currents to -3.0 ± 0.1 pA, 1 mM Ca^2+^ reduced it to -2.8 ± 0.1 pA and 1 mM Ba^2+^ reduced it to -3.2 ± 0.1 pA (Fig 4). The aforementioned values correspond to 0.75, 0.71 and 0.81 fold that of the divalent free K^+^ current for Mg^2+^, Ca^2+^ and Ba^2+^, respectively. Using the Woodhull model [18], the K_D_ of each divalent (effective binding to the pore) was calculated and in ascending order: Ca^2+^ (2.45 mM) >> Mg^2+^ (3.03 mM) >> Ba^2+^ (4.26 mM). At 10 μM and 100 μM divalents we observed smaller reductions (Fig 5A and 5B).

### Inhibition of K^+^ Fluxes by the Organic Cations: TMA (Tetramethyl Ammonium) and TEA (Tetraethyl Ammonium)

**Fig 6:**
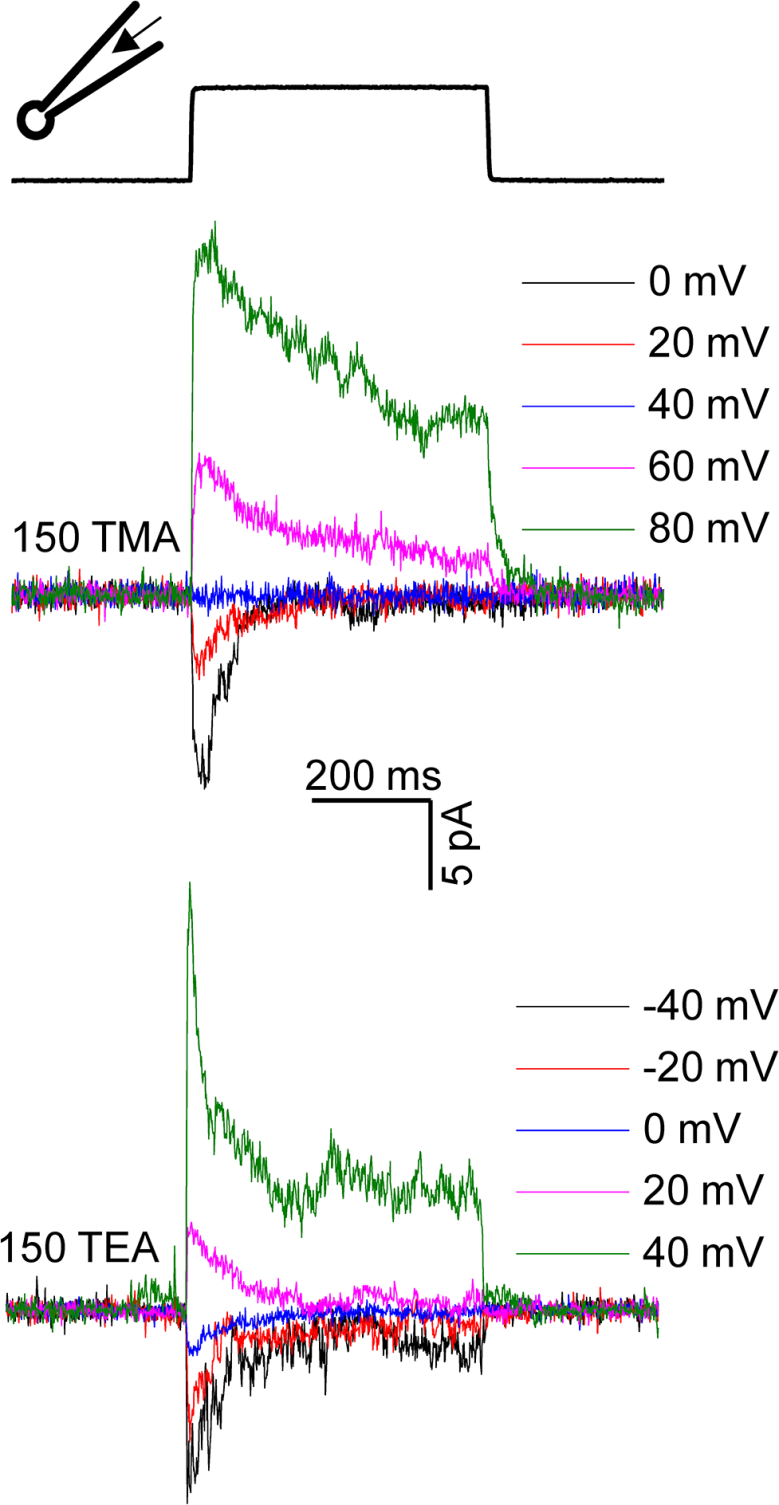
Permeation of TMA and TEA in outside-out patches. Current elicited from outside-out patches in response to 500 ms *positive* pressure pulses. Pipette solutions contained either 150 mM TMA or 150 mM TEA, 10 HEPES at pH 7.4. Divalent ions were not included in the pipette solution. For 150 mM TMA, the current would reverse near +45 mV, whereas with 150 mM TEA, the reversal potential would be near +8 mV (n = 3 patches) after compensating for the junction potential. The calculated junction potential was -5.0 mV for the 150 mM TMA containing pipette solution and -7.7 mV for the 150 mM TEA containing pipette solution.

Organic ions are larger than the univalent and divalent cations and are often used to provide an estimate of the dimensions of the pore. In cell-attached recordings with extracellular organic ions in the pipette we observed that: 1) the resistance of patches rapidly decreased at positive command (pipette) potentials (> +60 mV) (although it reversed immediately upon returning to negative command potentials), and 2) the amplitude of outward cytoplasmic K^+^ currents was much lower than what would be expected with K^+^ alone as the current carrier (data not shown). The first observation may be a result of variations in the patch seal resistance following changes in ionic composition [19]. In contrast, outside-out patches were stable with the cytoplasmic side exposed to 150 mM TMA or TEA and K^+^ bath saline on the extracellular side (Fig 6). TMA and TEA showed measurable outward current in outside-out patches indicating that these ions can permeate the channel and although permeating slowly could be displaced by an opposing K^+^ flux. Interestingly, the reversal potential of the current with TMA was around +40 mV (Fig 6, top) and the reversal potential with TEA was near ~ 0 mV (Fig 6, bottom). While the predicted reversal potential in these non-equilibrium conditions is undefined and we take these differences to reflect the relative permeability of the organic ions with the K^+^ efflux.

**Fig 7:**
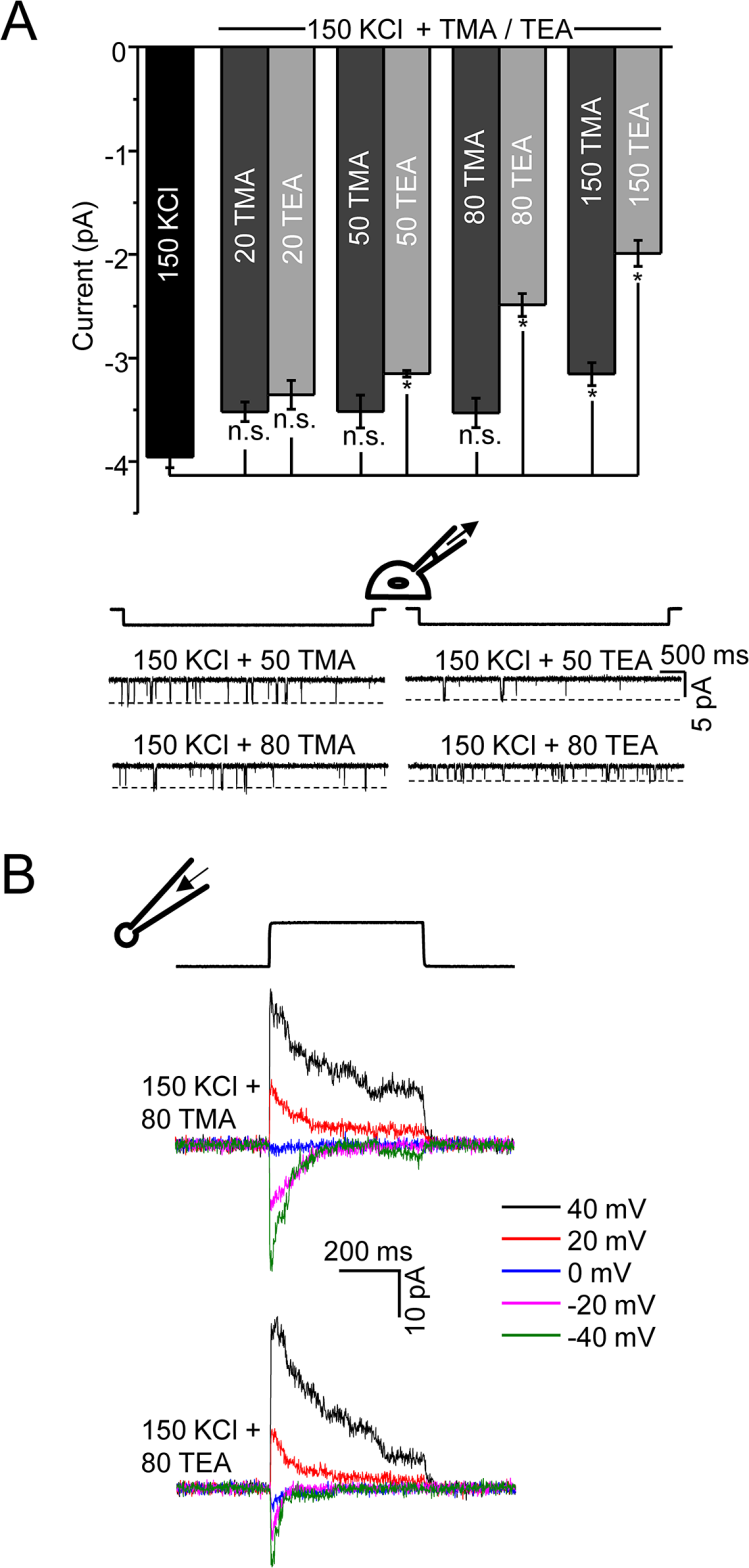
Titration of K^+^ currents by TMA and TEA. (A) Bar graph summarizing the effect of increasing concentrations of TMA or TEA (20 mM to 150 mM) on the unitary conductance when the main permeant ion was K^**+**^ in cell-attached patches. Student’s t-tests were performed to examine if reductions in current amplitudes (mean ± s.e.m) were statistically significant (*: P < 0.01; n.s.: not significant). As Fig 5 shows, both TMA and TEA can traverse the channel. The current traces shown below the bar graph (left: TMA, right: TEA) highlight the decrease in unitary current with TEA compared to TMA, suggesting TEA is the better blocker (n = 3 patches for each organic ion concentration). The junction potentials were ignored (for mixed solutions of TMA and TEA) because they were between -0.5mV and -3.7 mV for 20–150 mM TMA; for 20–150 mM TEA they were between -0.7 mV and -5.3 mV. (B) Current from outside-out patches with mixed solutions of K^**+**^ with either TMA or TEA (n = 3). The current is due to the permeation of both organic cations and K^**+**^. As expected, the amplitude of current produced with mixed solutions is lower compared to that with pure K^**+**^.

We titrated the K^+^ currents with TMA or TEA and observed a reduction of unitary current amplitude similar to that seen with the divalents (Fig 7A). For both TMA and TEA, the inhibition of unitary current was maximal with equimolar organic ion and K^+^ mixtures (150 mM each). At 150 mM, TMA reduced the K^+^ current at -80 mV to -3.5 ± 0.1 pA whereas TEA reduced it to -2.0 ± 0.1 pA (Fig 7A). As expected from a partial block of channels with TMA or TEA, we observed small currents in outside-out patches in the presence of a high concentration of these organic cations (150mM KCl mixed with 80 mM TMA or 80 mM TEA) (Fig 7B).

## Discussion

The principal findings of this study are that hPIEZO1 is permeable to monovalent ions (K^+^, Na^+^, Cs^+^, Li^+^) and most divalent ions (Ba^2+^, Ca^2+^ and Mg^2+^), but not to Mn^2+^. The organic ions TMA and TEA are weakly permeable. The alkali ions had similar conductances with the exception of Li^+^, which had markedly lower conductance. The permeability sequence of alkali ions was P_K_ > P_Cs_ ≅ P_Na_ > P_Li_ (1.0: 0.88: 0.82: 0.71). Given that the hydrated diameter of the ions differ, the sensitivity of the permeability sequence to Li^+^ suggests the effective pore diameter of the channel is larger (~ 8A˚) than the hydrated diameter of Li^+^. The permeabilities and conductances of Na^+^ and Cs^+^ were remarkably close suggesting that the channel has little ability to distinguish between these ions and the difference between the hydrated radii does not influence the conductance significantly since the pore is larger than both.

We measured unitary current amplitudes primarily in cell-attached patches which were more reliable than excised patches. For the purpose of comparison over concentration, we measured the unitary conductance at a consistent driving force of 40 mV. The conductance for K^+^ seemed to saturate at 150–300 mM in accordance with the expected [K^+^] versus unitary current relationship which is a hyperbolic increase to saturation. Although the limitation with cell-attached patches is that the voltage range over which conductance can be measured is limited to ~200 mV (membrane potential from -100 mV to +100 mV), we chose this configuration over the outside-out configuration which showed significant activity rundown. This voltage limitation might eventually be overcome by using channels reconstituted in synthetic bilayers where permeant ion concentration can be raised on both sides of the membrane as has been done with KcsA [20]. Since very low and very high concentrations are impractical to work with in the cell-attached configuration, we chose a more abbreviated range (10–300 mM) where we could obtain stable recordings.

Extracellular Ba^2+^, Ca^2+^ and Mg^2+^ permeate the channel at negative membrane potentials in cell-attached patches. However, for reasons we do not yet understand, fewer patches showed activity in the presence of 90 mM divalents compared to patches with K^+^ as the permeant ion. The high concentrations of divalents may have allosteric effects upon the channel and aside from other effects will reduce the surface potential [21–23]. The divalents, except for Mn^2+^ are permeant and whole-cell currents carried by Ca^2+^ and Mg^2+^ have been previously demonstrated [1]. Due to difficulties in activating the channels with high concentrations of divalents, we examined the ability of physiologically relevant concentrations of divalents (10 μM, 100 μM and 1mM) to reduce K^+^ currents.

The reduction in unitary K^+^ current was highest when Ca^2+^ was the titrating ion, followed by Mg^2+^ and then Ba^2+^. These data are consistent with low permeability of the divalents compared to K^+^. The reduction in K^+^ current reflects the fraction of the time that the divalent ions spend traversing the channel. Ca^2+^ ions permeate hPIEZO1 channels most slowly, i.e., they have a higher affinity for the channel. Mn^2+^ produced neither averaged currents nor single-channel currents suggesting that this ion is effectively impermeant. This difference from other divalents is presumably a result of the size and polarizability of the ion since the valence is the same. Mn^2+^ has a higher enthalpy of hydration than the other divalents and hence a larger hydration shell [3, 24].

The sizes of TMA and TEA are larger than those of the inorganic monovalent ions and their periphery is hydrophobic so they cannot form hydrogen bonds with residues lining the pore or permeating water molecules. Outside-out patches showed that TMA and TEA can slowly permeate the channel. TMA permeates somewhat more rapidly than TEA as reflected in the ~40 mV difference in reversal potentials. In agreement with the organic ion currents, the unitary current at negative potentials (in the cell-attached configuration) with solutions of K^+^ and TMA is higher than that with K^+^ and TEA because TEA is less permeable and hence more effective at blocking K^+^.

The channel pore appears wide enough for hydrated divalent ions such as Mg^2+^ and Ca^2+^ (hydrated radii of ~2 A˚ and ~2.45 A˚, respectively) to pass through slowly [25, 26]. The hydration energies of Mg^2+^ or Ca^2+^ (-476 and -397 kcal/mol, respectively at 25°C) ions suggest that their interactions with the hydration shell waters are strong, which will tend to retard permeation [3]. Monovalent alkali ions permeate the channel but the channel shows a slight inability to distinguish between Cs^+^ and Na^+^ suggesting shielding of these naked ions by water molecules. The capability of the channel to distinguish Li^+^ from other alkali ions suggests that the lower limit of the pore diameter is comparable to the hydrated diameter of Li^+^ (~6 A˚). The ability of TMA and TEA to permeate the channel suggests a pore diameter is slightly larger than ~ 8 A˚ (the diameter of TEA).

Recent studies have shown that MmPiezo1 transduces shear stress signals in endothelial cells and MmPiezo2 seems to play a role in noxious mechanosensation in sensory neurons [1, 27, 28]. Knock-out of MmPiezo1 severely impacts vascular development in mice [29, 30] and pathogenic mutations in hPIEZO1 lead to ion imbalances like those in the hemolytic anemia known as hereditary xerocytosis [16, 31, 32]. Mutations in hPIEZO2 are associated with Distal Arthrogryposes (DA type 3 also known as Gordon Syndrome and DA type 5) and Marden-Walker syndrome [33, 34]. However, very little is known about how mutated PIEZO proteins cause these disorders since mechanoprotection suppresses activity of endogenous PIEZO channels. The channel pore is wide enough for divalent ions such as Mg^2+^ and Ca^2+^ to pass through and Ca^2+^ entry through mechanosensitive channels is known to activate downstream processes [35–37]. The divalent effects on the permeation of monovalents may also be relevant to pathology where divalent concentrations are modified *in-situ*. In conclusion, knowledge of conduction and permeation properties is useful in understanding both normal channel function as well as channel dysfunction in disease.

## Supporting Information

S1 Table Calculation of Junction potentials for experiments using different bath and pipette solutions. Calculation of junction potentials were performed in Axon pCLAMP 10 electrophysiology software (Molecular Devices) using the junction potential calculator in the Clampex module. Based on the patch configuration either “intact patch” or “excised patch” were chosen. In the table below V_c_, V_p_, V_L_ and V_m_ are the resting membrane potential, command potential, junction potential and final membrane potential corrected for junction potential and patch configuration, respectively. The details used in the calculation: bath solution, pipette solution, configuration, junction potential and how it affects the membrane potential are provided in the table below.
(DOCX)

## References

[1] Coste B, Mathur J, Schmidt M, Earley TJ, Ranade S, Petrus MJ, et al Piezo1 and Piezo2 are essential components of distinct mechanically activated cation channels. Science. 2010;330(6000):55–60. 10.1126/science.1193270 20813920 PMC3062430

[2] Coste B, Xiao B, Santos JS, Syeda R, Grandl J, Spencer KS, et al Piezo proteins are pore-forming subunits of mechanically activated channels. Nature. 2012;483(7388):176–81. 10.1038/nature10812 22343900 PMC3297710

[3] Hille B. Ion channels of Excitable Membranes (Sinauer Associates, Inc). 2001:441–70.

[4] Akk G, Auerbach A. Inorganic, monovalent cations compete with agonists for the transmitter binding site of nicotinic acetylcholine receptors. Biophysical journal. 1996;70(6):2652–8. 10.1016/S0006-3495(96)79834-X 8744302 PMC1225244

[5] Ding S, Sachs F. Ion permeation and block of P2X(2) purinoceptors: single channel recordings. The Journal of membrane biology. 1999;172(3):215–23. . 10568791 10.1007/s002329900598

[6] Hess P, Lansman JB, Tsien RW. Calcium channel selectivity for divalent and monovalent cations. Voltage and concentration dependence of single channel current in ventricular heart cells. The Journal of general physiology. 1986;88(3):293–319. 2428919 10.1085/jgp.88.3.293 PMC2228831

[7] Besch SR, Suchyna T, Sachs F. High-speed pressure clamp. Pflugers Archiv: European journal of physiology. 2002;445(1):161–6. 10.1007/s00424-002-0903-0 . 12397401

[8] Nicolai C, Sachs F. Solving ion channel kinetics with the QuB software. Biophysical Reviews and Letters. 2013;08(03n04):191–211. 10.1142/S1793048013300053

[9] Adams DJ, Dwyer TM, Hille B. The permeability of endplate channels to monovalent and divalent metal cations. The Journal of general physiology. 1980;75(5):493–510. 6247423 10.1085/jgp.75.5.493 PMC2215258

[10] Burnashev N, Zhou Z, Neher E, Sakmann B. Fractional calcium currents through recombinant GluR channels of the NMDA, AMPA and kainate receptor subtypes. The Journal of physiology. 1995;485 (Pt 2):403–18. 7666365 10.1113/jphysiol.1995.sp020738 PMC1158001

[11] Decker ER, Dani JA. Calcium permeability of the nicotinic acetylcholine receptor: the single-channel calcium influx is significant. The Journal of neuroscience: the official journal of the Society for Neuroscience. 1990;10(10):3413–20. . 2170596 10.1523/JNEUROSCI.10-10-03413.1990 PMC6570188

[12] Evans RJ, Lewis C, Virginio C, Lundstrom K, Buell G, Surprenant A, et al Ionic permeability of, and divalent cation effects on, two ATP-gated cation channels (P2X receptors) expressed in mammalian cells. The Journal of physiology. 1996;497 (Pt 2):413–22. 8961184 10.1113/jphysiol.1996.sp021777 PMC1160993

[13] Villarroel A, Burnashev N, Sakmann B. Dimensions of the narrow portion of a recombinant NMDA receptor channel. Biophysical journal. 1995;68(3):866–75. 10.1016/S0006-3495(95)80263-8 7538803 PMC1281811

[14] Gottlieb PA, Bae C, Sachs F. Gating the mechanical channel Piezo1: a comparison between whole-cell and patch recording. Channels. 2012;6(4):282–9. 10.4161/chan.21064 22790451 PMC3508907

[15] Morris CE. Mechanoprotection of the plasma membrane in neurons and other non-erythroid cells by the spectrin-based membrane skeleton. Cellular & molecular biology letters. 2001;6(3):703–20. . 11598643

[16] Bae C, Gnanasambandam R, Nicolai C, Sachs F, Gottlieb PA. Xerocytosis is caused by mutations that alter the kinetics of the mechanosensitive channel PIEZO1. Proceedings of the National Academy of Sciences of the United States of America. 2013;110(12):E1162–8. 10.1073/pnas.1219777110 23487776 PMC3606986

[17] Bae C, Sachs F, Gottlieb PA. The mechanosensitive ion channel Piezo1 is inhibited by the peptide GsMTx4. Biochemistry. 2011;50(29):6295–300. 10.1021/bi200770q 21696149 PMC3169095

[18] Woodhull AM. Ionic blockage of sodium channels in nerve. The Journal of general physiology. 1973;61(6):687–708. 4541078 10.1085/jgp.61.6.687 PMC2203489

[19] Suchyna TM, Markin VS, Sachs F. Biophysics and structure of the patch and the gigaseal. Biophysical journal. 2009;97(3):738–47. 10.1016/j.bpj.2009.05.018 19651032 PMC2718145

[20] Kutluay E, Roux B, Heginbotham L. Rapid intracellular TEA block of the KcsA potassium channel. Biophysical journal. 2005;88(2):1018–29. 10.1529/biophysj.104.052043 15556975 PMC1305109

[21] Zhang PC, Keleshian AM, Sachs F. Voltage-induced membrane movement. Nature. 2001;413(6854):428–32. 10.1038/35096578 . 11574890

[22] Golowasch J, Kirkwood A, Miller C. Allosteric effects of Mg2+ on the gating of Ca2+-activated K+ channels from mammalian skeletal muscle. The Journal of experimental biology. 1986;124:5–13. . 2428908 10.1242/jeb.124.1.5

[23] McLaughlin SG, Szabo G, Eisenman G. Divalent ions and the surface potential of charged phospholipid membranes. The Journal of general physiology. 1971;58(6):667–87. 5120393 10.1085/jgp.58.6.667 PMC2226047

[24] Burgess J. Ions in Solution: Basic Principles of Chemical Interactions. (Prentice Hall Professional Technical Reference. 1988.

[25] Bruni F, Imberti S, Mancinelli R, Ricci MA. Aqueous solutions of divalent chlorides: ions hydration shell and water structure. The Journal of chemical physics. 2012;136(6):064520 10.1063/1.3684633 . 22360208

[26] Marcus Y. Thermodynamics of solvation of ions. Part 5.-Gibbs free energy of hydration at 298.15 K. Journal of the Chemical Society, Faraday Transactions. 1991;87(18):2995–9. 10.1039/FT9918702995

[27] Dubin AE, Schmidt M, Mathur J, Petrus MJ, Xiao B, Coste B, et al Inflammatory signals enhance piezo2-mediated mechanosensitive currents. Cell reports. 2012;2(3):511–7. 10.1016/j.celrep.2012.07.014 22921401 PMC3462303

[28] Kim SE, Coste B, Chadha A, Cook B, Patapoutian A. The role of Drosophila Piezo in mechanical nociception. Nature. 2012;483(7388):209–12. 10.1038/nature10801 22343891 PMC3297676

[29] Li J, Hou B, Tumova S, Muraki K, Bruns A, Ludlow MJ, et al. Piezo1 integration of vascular architecture with physiological force. Nature. 2014. 10.1038/nature13701 . 25119035 PMC4230887

[30] Ranade SS, Qiu Z, Woo SH, Hur SS, Murthy SE, Cahalan SM, et al Piezo1, a mechanically activated ion channel, is required for vascular development in mice. Proceedings of the National Academy of Sciences of the United States of America. 2014;111(28):10347–52. 10.1073/pnas.1409233111 24958852 PMC4104881

[31] Archer NM, Shmukler BE, Andolfo I, Vandorpe DH, Gnanasambandam R, Higgins JM, et al. Hereditary xerocytosis revisited. American journal of hematology. 2014. 10.1002/ajh.23799 . 25044010 PMC4237618

[32] Albuisson J, Murthy SE, Bandell M, Coste B, Louis-Dit-Picard H, Mathur J, et al Dehydrated hereditary stomatocytosis linked to gain-of-function mutations in mechanically activated PIEZO1 ion channels. Nature communications. 2013;4:1884 10.1038/ncomms2899 23695678 PMC3674779

[33] Coste B, Houge G, Murray MF, Stitziel N, Bandell M, Giovanni MA, et al Gain-of-function mutations in the mechanically activated ion channel PIEZO2 cause a subtype of Distal Arthrogryposis. Proceedings of the National Academy of Sciences of the United States of America. 2013;110(12):4667–72. 10.1073/pnas.1221400110 23487782 PMC3607045

[34] McMillin MJ, Beck AE, Chong JX, Shively KM, Buckingham KJ, Gildersleeve HI, et al Mutations in PIEZO2 cause Gordon syndrome, Marden-Walker syndrome, and distal arthrogryposis type 5. American journal of human genetics. 2014;94(5):734–44. 10.1016/j.ajhg.2014.03.015 24726473 PMC4067551

[35] Hoyer J, Distler A, Haase W, Gogelein H. Ca2+ influx through stretch-activated cation channels activates maxi K+ channels in porcine endocardial endothelium. Proceedings of the National Academy of Sciences of the United States of America. 1994;91(6):2367–71. 7510889 10.1073/pnas.91.6.2367 PMC43372

[36] McCarter GC, Levine JD. Ionic basis of a mechanotransduction current in adult rat dorsal root ganglion neurons. Molecular pain. 2006;2:28 10.1186/1744-8069-2-28 16923187 PMC1563451

[37] Sigurdson W, Ruknudin A, Sachs F. Calcium imaging of mechanically induced fluxes in tissue-cultured chick heart: role of stretch-activated ion channels. The American journal of physiology. 1992;262(4 Pt 2):H1110–5. . 1373571 10.1152/ajpheart.1992.262.4.H1110

