# Supplementary material for: Ionic Selectivity and Permeation Properties of Human PIEZO1 Channels
Source: PLoS One. 2015 May 8;10(5):e0125503. doi: 10.1371/journal.pone.0125503 (PMC4425559; doi:10.1371/journal.pone.0125503)
Supplement: S1 Table — Calculation of junction potentials were performed in Axon pCLAMP 10 electrophysiology software (Molecular Devices) using the junction potential calculator in the Clampex module. Based on the patch configuration either “intact patch” or “excised patch” were chosen. In the table below Vc, Vp, VL and Vm are the resting membrane potential, command potential, junction potential and final membrane potential corrected for junction potential and patch configuration, respectively. The details used in the calculation: bath solution, pipette solution, configuration, junction potential and how it affects the membrane potential are provided in the table below. (DOCX) [file pone.0125503.s001.docx]

**Supporting information**

**S1 Table. Calculation of Junction potentials for experiments using different bath and pipette solutions**

| **Bath solutions** | **Pipette solutions** | **Formula for Vm caluclation** | **Junc. Potential (V_L_)** | **Membrane potential (V_m_)** |
| --- | --- | --- | --- | --- |
| 150 KCl, 1MgCl_2_, 1CaCl_2_, 10 HEPES, pH 7.4 |  | OO patch  (V_m_=V_p_-V_L_) |  |  |
|  | 150 NaCl, 10 HEPES, pH 7.4 |  | -4.0 | Vp+4.0 mV |
|  | 150 KCl, 10 HEPES, pH 7.4 |  | 0.2 | Vp-0.2 mV |
|  | 150 CsCl, 10 HEPES, pH 7.4 |  | 0.8 | Vp-0.8 mV |
|  | 150 LiCl, 10 HEPES, pH 7.4 |  | -6.4 | Vp+6.4 mV |
|  |  |  |  |  |
| 150 KCl, 1MgCl_2_, 1CaCl_2_, 10 HEPES, pH 7.4 |  | OO patch  (V_m_=V_p_-V_L_) |  |  |
|  | 150 TMA, 10 HEPES, pH 7.4 |  | -5.0 | Vp+5.0 mV or ‑Vp+5.0 mV |
|  | 150 TEA, 10 HEPES, pH 7.4 |  | -7.7 | Vp+7.7 mV or ‑Vp+7.7 mV |
|  |  |  |  |  |
| 150 KCl, 1MgCl_2_, 1CaCl_2_, 10 HEPES, pH 7.4 |  | OO patch  (V_m_=V_p_-V_L_) |  |  |
|  | 150 KCl, 80 TMA, 10 HEPES, pH 7.4 |  | -2.2 | Vp+2.2 mV |
|  | 150 KCl, 80 TEA, 10 HEPES, pH 7.4 |  | -3.1 | Vp+3.1 mV |
|  |  |  |  |  |
| 150 KCl, 1MgCl_2_, 1CaCl_2_, 10 HEPES, pH 7.4 |  | CA patch  V_m_=(V_c_‑V_p_)+(V_L_) |  |  |
|  | 10 KCl, 10 HEPES, pH 7.4 |  | 1.8 | -Vp+1.8 mV |
|  | 30 KCl, 10 HEPES, pH 7.4 |  | 1.2 | -Vp+1.2 mV |
|  | 70 KCl, 10 HEPES, pH 7.4 |  | 0.7 | -Vp+0.7 mV |
|  | 100 KCl, 10 HEPES, pH 7.4 |  | 0.5 | -Vp+0.5 mV |
|  | 150 KCl, 10 HEPES, pH 7.4 |  | 0.2 | -Vp+0.2 mV |
|  | 300 KCl, 10 HEPES, pH 7.4 |  | -0.2 | -Vp-0.2 mV |
|  |  |  |  |  |
| 150 KCl, 1MgCl_2_, 1CaCl_2_, 10 HEPES, pH 7.4 |  | CA patch  V_m_=(V_c_-V_p_)+(V_L_) |  |  |
|  | 90 BaCl_2_, 10 HEPES, pH 7.4 |  | -7.8 | -Vp-7.8 mV |
|  | 90 CaCl_2_, 10 HEPES, pH 7.4 |  | -8.3 | -Vp-8.3 mV |
|  | 90 MgCl_2_, 10 HEPES, pH 7.4 |  | -9.1 | -Vp-9.1 mV |
|  |  |  |  |  |
| 150 KCl, 1MgCl_2_, 1CaCl_2_, 10 HEPES, pH 7.4 |  | CA patch  V_m_=(V_c_-V_p_)+(V_L_) |  |  |
|  | 150 KCl, 20 TMA, 10 HEPES, pH 7.4 |  | -0.5 | -Vp-0.5 mV |
|  | 150 KCl, 50 TMA, 10 HEPES, pH 7.4 |  | -1.4 | -Vp-1.4 mV |
|  | 150 KCl, 80 TMA, 10 HEPES, pH 7.4 |  | -2.2 | -Vp-2.2 mV |
|  | 150 KCl, 150 TMA, 10 HEPES, pH 7.4 |  | -3.7 | -Vp-3.7 mV |
|  | 150 KCl, 20 TEA, 10 HEPES, pH 7.4 |  | -0.7 | -Vp-0.7 mV |
|  | 150 KCl, 50 TEA, 10 HEPES, pH 7.4 |  | -2.0 | -Vp-2.0 mV |
|  | 150 KCl, 80 TEA, 10 HEPES, pH 7.4 |  | -3.1 | -Vp-3.1 mV |
|  | 150 KCl, 150 TEA, 10 HEPES, pH 7.4 |  | -5.3 | -Vp-5.3 mV |
|  |  |  |  |  |
| 150 KCl, 1MgCl_2_, 1CaCl_2_, 10 HEPES, pH 7.4 |  | CA patch  V_m_=(V_c_-V_p_)+(V_L_) |  |  |
|  | 150 KCl, 1BaCl_2_, 10 HEPES, pH 7.4 |  | 0.5 | -Vp+0.5 mV |
|  | 150 KCl, 1CaCl_2_, 10 HEPES, pH 7.4 |  | 0.3 | -Vp+0.3 mV |
|  | 150 KCl, 1MgCl_2_, 10 HEPES, pH 7.4 |  | 0.3 | -Vp+0.3 mV |
|  |  |  |  |  |
